# A 3 MHz Low-Error Adaptive Howland Current Source for High-Frequency Bioimpedance Applications

**DOI:** 10.3390/s24134357

**Published:** 2024-07-04

**Authors:** Ifeabunike I. Nwokoye, Iasonas F. Triantis

**Affiliations:** Research Centre for Biomedical Engineering, City, University of London, London EC1V 0HB, UK; ifeabunike.nwokoye@city.ac.uk

**Keywords:** bioimpedance, bioelectrical impedance, electrical impedance, impedance spectroscopy, Howland current source, AC current source

## Abstract

Bioimpedance is a diagnostic sensing method used in medical applications, ranging from body composition assessment to detecting skin cancer. Commonly, discrete-component (and at times integrated) circuit variants of the Howland Current Source (HCS) topology are employed for injection of an AC current. Ideally, its amplitude should remain within 1% of its nominal value across a frequency range, and that nominal value should be programmable. However, the method’s applicability and accuracy are hindered due to the current amplitude diminishing at frequencies above 100 kHz, with very few designs accomplishing 1 MHz, and only at a single nominal amplitude. This paper presents the design and implementation of an adaptive current source for bioimpedance applications employing automatic gain control (AGC). The “Adaptive Howland Current Source” (AHCS) was experimentally tested, and the results indicate that the design can achieve less than 1% amplitude error for both 1 mA and 100 µA currents for bandwidths up to 3 MHz. Simulations also indicate that the system can be designed to achieve up to 19% noise reduction relative to the most common HCS design. AHCS addresses the need for high bandwidth AC current sources in bioimpedance spectroscopy, offering automatic output current compensation without constant recalibration. The novel structure of AHCS proves crucial in applications requiring higher β-dispersion frequencies exceeding 1 MHz, where greater penetration depths and better cell status assessment can be achieved, e.g., in the detection of skin or breast cancer.

## 1. Introduction

Bioelectrical impedance or *bioimpedance* measurements (BIMs) involve injecting a very low alternating electric current into a biological tissue sample and directly deriving the sample’s impedance from the measurement of the resulting voltage [1,2]. This technique has been established across a wide range of applications, including lung disease diagnosis and detection (Electrical Impedance Tomography—EIT) [3], cancerous tissue characterisation and segregation [4], neuromuscular disease assessment [5], cardiac output monitoring [6], body composition analysis [7], knee monitoring [8], and food quality assessment [9], to name a few. 

The accuracy of bioimpedance measurements heavily relies on that of the front-end instrumentation [10,11], which comprises two main stages, the current injection and the voltage measurement circuitry. The design of the latter can vary, with common topologies including *synchronous detection* (or demodulation) [12], *synchronous sampling* [13], or *magnitude and phase* [14]. On the other hand, the design of the current injection instrumentation usually involves an AC current source comprising a signal generator connected to a voltage-to-current converter [15,16]. For precise BIM, the current source must deliver a constant-amplitude (less than 1% amplitude variability [17,18]) AC current over a wide range of loads, typically from hundreds of Ω to tens of kΩ—respectively requiring injected currents of a few mA down to tens of µA (sub-mA). Desired bandwidths range from 1 kHz to several MHz; however, nonidealities degrade the output impedance of current sources at high frequencies [19]. Consequently, the output current amplitude drops with frequency, relative to its intended nominal value, resulting in considerable accuracy degradation. This reduction in output current is herein termed (and also in [18]) as “current amplitude error” or simply “*current error*”. 

### 1.1. Current Source Topologies

With the exception of some application-specific integrated circuit (ASIC) realisations (e.g., [18]), the majority of bioimpedance circuitry makes use of discrete component versions of the Howland Current Source (HCS) [20,21], which is a voltage-controlled current source (VCCS) topology. In the last few decades, several HCS variants have been suggested to improve the initial design’s performance, mostly aiming to improve output impedance so as to avoid current fluctuations with frequency and load impedance [20]. 

**Figure 1:**
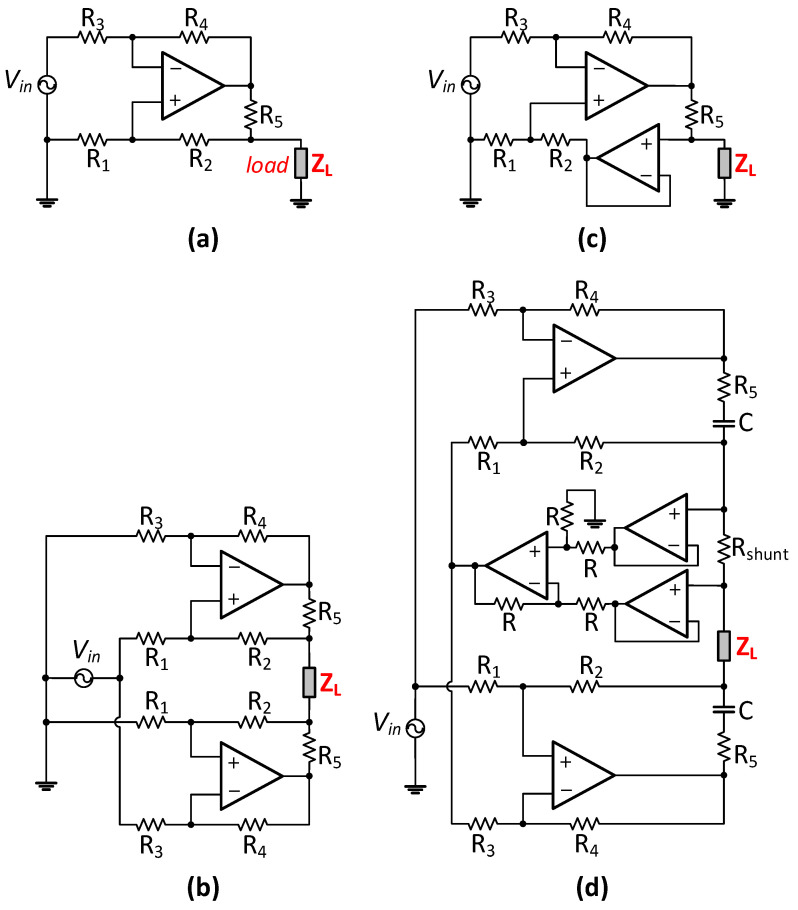
Some evolutionary topologies of the Howland Current Source (HCS) for enhanced output swing (**a**,**b**), enhanced output impedance (**c**), and higher bandwidth (**d**), specifically showcasing (**a**) Enhanced HCS (EHCS) [20], (**b**) Mirrored Enhanced Howland Current Source (MEHCS) [15], (**c**) EHCS with buffered feedback [19], and (**d**) MEHCS with feedback control [22].

The Enhanced Howland Current Source (EHCS) in Figure 1a achieves stability and high-output impedance through precise matching of the feedback resistors, with the transconductance determined by R_5_. While it enhances output voltage swing compared to the basic HCS, its grounded load configuration is undesirable for biomedical applications. Moreover, like other topologies, it still exhibits output impedance degradation.

The Mirrored Enhanced Howland Current Source (MEHCS, Figure 1b) is widely considered the best performing topology due to its true differential output and its overall simplicity and robustness relative to other designs [15,22]. Still, like all open-loop current sources, MEHCS also has drawbacks, including feedback resistor mismatches; operational amplifier limitations; stray capacitances in tracks and cables; and other issues degrading its output impedance, with the topology exhibiting more than 1% current error for bandwidths over a few hundred kHz [16]. The buffered feedback topology in Figure 1c features the same transconductance as EHCS. A buffer added in the positive feedback of the current source improves the matching of resistors R_1-4_ and increases the output impedance, albeit not very significantly [19,22]. This topology is more appropriate for grounded load applications, as adopting it in a mirrored topology further increases mismatches, increases common mode error, and reduces output swing.

Other design variations that have been made towards improving the HCS output impedance include using a general impedance converter (GIC) [17] or a negative impedance converter (NIC) [23]. However, the improvements made by these circuits are very limited, with no adaptability, as NIC and GIC do not work at all frequencies and need recalibration for every frequency change [17,22]. One of the better performing designs, the NIC Tietze circuit [24], achieved a reported 1 MHz bandwidth; however, the current error was 2.33% and thus higher than 1%.

The current-to-voltage feedback topology [22] (CTVF-MEHCS—Figure 1d) features similar transconductance to MEHCS. It compensates for leakage current by sensing the output current and providing negative feedback to the MEHCS input, thereby effectively increasing the output impedance at higher frequencies. Experimental results employing this technique reported approximately a 1.5% error for a 1mA current amplitude over a 1MHz bandwidth, a significant improvement over MEHCS. However, its suitability for sub-mA output current is compromised, as accuracy degrades when generating lower current amplitudes. Additionally, the resistor matching requirements are greater than MEHCS [22].

### 1.2. Need for Higher Bandwidth

At frequencies near and above 1 MHz, the tissue properties monitored are associated mainly with intracellular structures like cell membranes and organelles (part of β-dispersion bioimpedance frequency band) [1,25]. Being able to monitor biological tissue accurately at frequencies above the abovementioned instrumentation limitations is crucial in bioimpedance applications like skin, oral, and breast cancer detection and assessment [26,27]. Still, to the authors’ knowledge the “barrier” of sub-1% error over 1 MHz for mA and sub-mA current amplitudes has not yet been surpassed. 

To overcome this barrier, the work presented here introduces and experimentally evaluates a MEHCS-based automatic gain control (AGC) design offering a high-bandwidth (3 MHz) adaptive current source achieving significantly low amplitude error. We term this the “Adaptive Howland Current Source” (AHCS), and we implement it in a discrete-component realisation, in line with the majority of the relevant literature [20]. This novel design adjusts the gain of the current driver automatically, to maintain the current output’s accuracy, regardless of any output impedance degradation.

## 2. Methods

### 2.1. AGC Architecture

**Figure 2:**
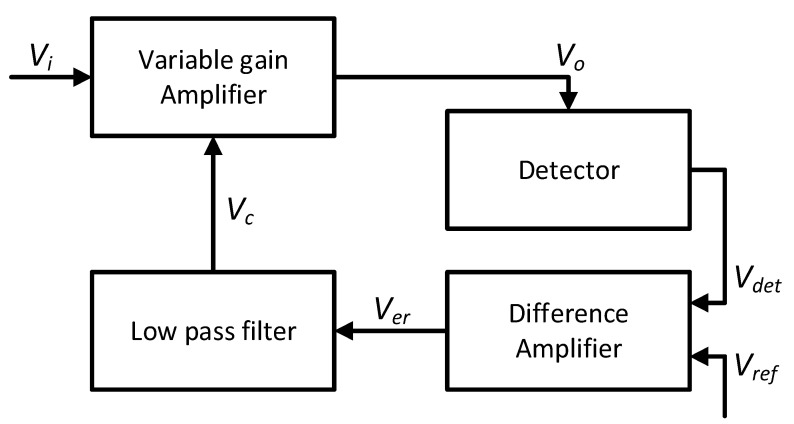
AGC system for amplitude stabilisation [29].

The system presented here employs a standard automatic gain control (AGC) mechanism for amplitude stabilisation, utilising a variable gain amplifier (VGA). In a typical VGA-based AGC loop—like that in Figure 2—the input signal *V_i_* passes through the VGA to produce the output level *V_o_* to be stabilised [28]. The detector’s output (*V_det_*) is compared against a desired output amplitude reference voltage (*V_ref_*), producing an error signal (*V_er_*). That is then integrated, producing a gain control feedback voltage (V_c_) and dynamically adjusting the VGA gain, thus stabilising the output to an amplitude (*V_o_*) that tracks *V_ref_*.

### 2.2. System Design

**Figure 3:**
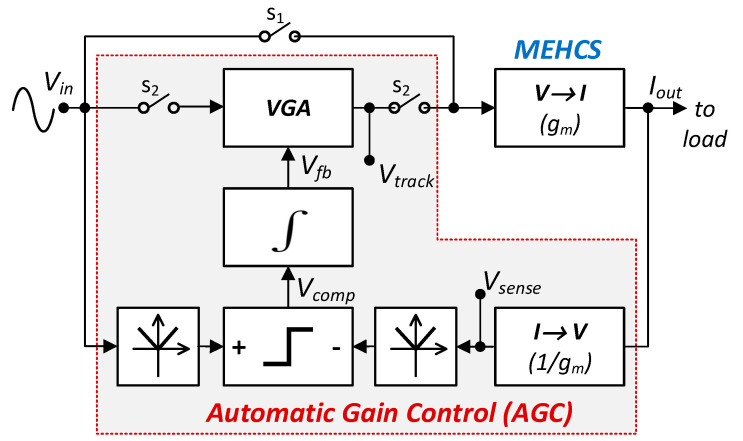
Functional block diagram of the AHCS.

As shown in the block diagram in Figure 3, with switches s_1_ on and s_2_ off, the system operates as an open-loop MEHCS with a transconductance g_m_ converting the input sinusoidal voltage *V*_in_(t) to output current *I*_out_(t). As mentioned, its amplitude should ideally be constant to a nominal value *I*_out_, irrespective of its frequency. However, as its amplitude drops due to the leakage current *I*_leak_ “lost” through the output impedance—which decays at higher frequencies—the output current becomes *I*_outER_ < *I*_out_ (Equation (1)):

(1) $$I_{outER}=I_{out}-I_{leak}$$

Once the automatic gain control (AGC) stage is on (s_1_ off, s_2_ on), the output current *I*_out_(t) of the MEHCS is continuously monitored through a transimpedance stage with a gain of 1/g_m_, whose output amplitude is ideally *V*_sense_ = *I*_out_/g_m_ = *V*_in_ if the output current has the nominal value *I*_out_. When the output current amplitude changes, *V*_sense_ = *I*_outER_/g_m_ ≠ *V*_in_. The gain *G*_COR_ by which the present output *I*_outER_ needs to be multiplied to achieve the nominal output current *I*_out_ is given by Equation (2):

(2) $$G_{COR}=\frac{I_{out}}{I_{outER}}=\frac{I_{out}}{I_{out}-I_{leak}}=\frac{\frac{I_{out}}{g_{m}}}{\frac{I_{out}-I_{leak}}{g_{m}}}=\frac{V_{in}}{V_{sense}}$$

To compare their values, peak detectors extracting the amplitudes *V*_sense_ and *V*_in_ are connected differentially to a comparator. As indicated in Equation (3), its output V_comp_(t) assumes a “high” or a “low” state, represented respectively by a positive (+V_settle_) or a negative (−V_settle_) DC voltage, depending on which of the inputs is higher. Once the inputs become equal, the comparator generates an oscillatory output at double the frequency of the injected signal, which is the dominant ripple frequency at the output of the peak detectors [29].

(3) $$V_{comp}\left(t\right)=\left\{\begin{array}{c} {+V}_{settle,}{\;V}_{sense}<V_{in}\;\left(comparator\;output\;high\;DC\right)\\ {-V}_{settle,\;}V_{sense}>V_{in}\;\left(comparator\;output\;low\;DC\right)\\ V_{osc}\left(t\right),\;V_{in}=V_{sense}\;\left(comparator\;output\;oscilates\right) \end{array}\right.$$

The oscillatory output of the comparator can be ideally approximated as a square wave with amplitude V_settle_ and frequency ω_x_. The Fourier series (up to 3rd harmonic) gives Equation (4):

(4) $$V_{cosc}\left(t\right)=\frac{4V_{settle}}{\pi }\left(sin\left(\omega_{x}t\right)+\frac{1}{3}sin\left(3\omega_{x}t\right)+\frac{1}{5}sin\left(5\omega_{x}t\right)+\frac{1}{7}sin\left(7\omega_{x}t\right)\right)$$

The comparator is connected to an integrator with high time constant τ. The integrator’s time constant is designed to be much higher than 1/ωx, assuming the dominant frequency of the comparator output oscillation when inputs are equal is the same as that of the injected signal or higher. The integrator’s output V_fb_(t) is described by Equation (5), where V_fb0_ is the integrator initial output at t = 0, assumed to be zero.

(5) $$V_{fb}\left(t\right)=\frac{1}{\tau }\int V_{comp}\left(t\right)dt+V_{fb0}$$

When V_comp_(t) assumes its high or low DC value, the integrator output will respectively be a positive or a negative ramp given by Equation (6).

(6) $$V_{fb}(t)=\left\{\begin{array}{c} \frac{V_{settle}}{\tau }t,\;\;integrator\;outputing\;a+ive\;ramp\\ \frac{V_{settle}}{\tau }t,\;integrator\;outputing\;a-ive\;ramp\; \end{array}\right.$$

In the case examined here, *V*_sense_ < *V*_in_ and the integrator will be a positive ramp. The integrator output controls the gain of a variable gain amplifier (VGA), whose initial gain is unity. Consequently, the amplitude of the VGA’s output *V*_track_ can be expressed using Equation (7):

(7) $$V_{track}\left(t\right)=V_{in}\left(t\right)\left(1+A_{vfb}\left(t\right)\right)$$

where *A*_vfb_(t) = V_fb_(t)/1V is the integrator output expressed as unitless gain. 

Thus, for *I*_outER_ < *I*_out_, the amplitude of the MEHCS input voltage (now *V*_track_) will ramp up in value until the transimpedance output amplitude reaches a value equal to *V*_in_. At that point, the comparator will start oscillating (Equation (3)), resulting in the integrator output shown in Equation (8), comprised of a high frequency triangular wave (due to the square wave nature of Equation (4)) around a dc value V_fbDC_:

(8) $$\begin{array}{c} V_{fb}\left(t\right)=\\ V_{fbDC}-\frac{4V_{settle}T_{x}}{2\pi^{2}\tau }\left(cos\left(\omega_{x}t\right)+\frac{1}{9}cos\left(3\omega_{x}t\right)+\frac{1}{25}cos\left(5\omega_{x}t\right)+\frac{1}{49}cos\left(7\omega_{x}t\right)\right) \end{array}$$

Given that τ >> T_x_, the triangular wave can be considered a ripple of negligible amplitude, and therefore the integrator output will settle at DC voltage *V*_fbDC_, whose unitless value expressed as gain is *A*_vfbDC_. Replacing *A*_vfb_(t) in Equation (7) with *A*_vfbDC_ and dividing both sides by *g*_m_, the input to MEHCS will now settle to a *V*_track_ value that will generate the desirable *I*_out_ rather than *I*_outER_ generated by the open-loop MEHCS as given by Equation (9) (expressed as amplitudes):

(9) $$\begin{array}{c} I_{out}=I_{outER}\left(1+A_{vfbDC}\right)\Rightarrow \\ \Rightarrow 1+A_{vfbDC}=\frac{I_{out}}{I_{out}-I_{leak}}\Rightarrow \\ \Rightarrow 1+A_{vfbDC}=G_{COR} \end{array}$$

The AGC ensures that the integrator output and thus the VGA gain will settle to the value necessary for compensating the current error through scaling the input voltage to the MEHCS, rather than by attempting to increase the output impedance as seen in most of the aforementioned topologies. Attempting to compensate by somehow adding *I*_leak_ is also not straightforward, as it would have to be AC and precisely in phase with *I*_out_.

### 2.3. Circuit Design

**Figure 4:**
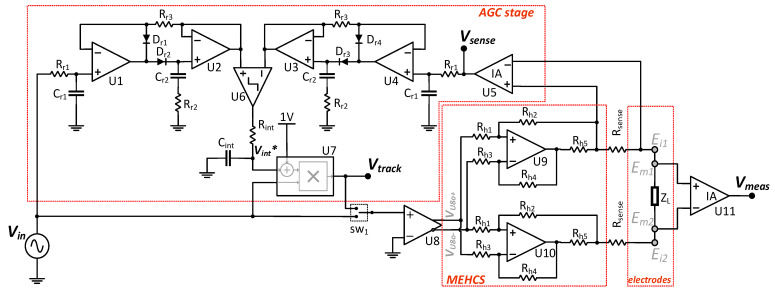
Schematic diagram of the AHCS circuit.

During the design phase of the AHCS circuit, critical factors, including feedback resistor network tolerances, op-amp matching (same die amps) and open-loop gain, and PCB layout were considered to ensure a desirable output. In the implementation of the AHCS circuit in Figure 4, the MEHCS stage is comprised of U9 and U10 op-amps, with its input driven by a unity gain single-to-differential amplifier (U8) to increase common mode rejection, reduce DC offsets, and thus enhance load capability [30]. In the open loop (sw_1_ to *V*_in_), the output current *I*_out_, flowing through *R*_sense_ and through the load Z_L_, is given by

(10) $$I_{out}=g_{m}V_{in}$$

where g_m_ is the transconductance of the MEHCS, given by [11]

(11) $$g_{m}=\frac{R_{h4}}{R_{h3}\times R_{h5}}$$

In MECHS, achieving high-output impedance requires *R_h_*_1_ to *R_h_*_4_ to be highly matched, high-value resistors [15] (in the range of 0.1–1 MΩ). Here, 0.01% tolerance 100 kΩ resistors were used. With the feedback on, the output current is continuously monitored via sensing resistor *R*_sense_ (inserted twice for output symmetry) and the sensing instrumentation amplifier U5, with gain *A*_v_sense_, designed so that its output equals the MEHCS input voltage:

(12) $$A_{v\_sense}=\frac{1}{g_{m}*R_{sense}}$$

The output current is now derived by replacing *V*_in_ with *V*_track_ in (10). *V*_track_ is the output of the AGC stage, comprised of two precision peak detectors (U_1,2_ and U_3,4_ combined with R_r1–3_, C_r1,2_, and D_1-4_); a comparator (U6); an integrator (*R*_int_, *C*_int_); and a VGA realised through an AD734 multiplier, with the base gain set to unity through a 1V DC reference to avoid a multiplication by zero. The AGC continuously monitors and compares the peak values of both *V_in_* and *V_sense_*. If the amplitude of *I*_out_ (i.e., *V_sense_*) starts dropping, the comparator output becomes high, causing the integrator output to ramp up, effectively increasing *V*_track_. Once the feedback achieves *V*_sense_ = *V*_in_, the comparator starts oscillating, causing *V*_int_ to stabilise to a settled value. The measured voltage *V_meas_* across the load *Z_L_* is given by U11, the measuring instrumentation amplifier, with a gain of *A_V_meas_*.

### 2.4. Experimental Setup

**Figure 5:**
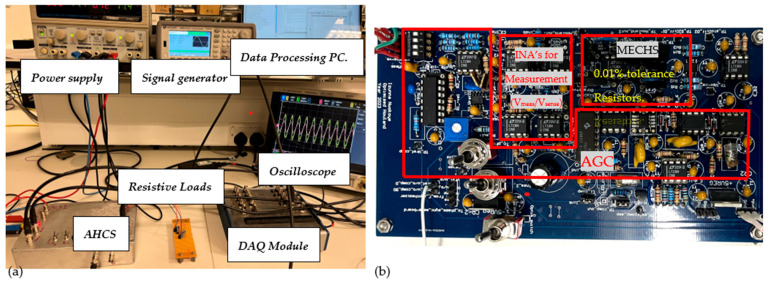
(**a**) Photograph of the AHCS experimental setup. (**b**) Photograph of the AHCS circuit board with the main stages labelled.

The experimental setup in Figure 5a was configured to assess the performance of AHCS for a range of loads and frequencies. The setup features a power supply, a signal generator to generate the *V*_in_, an oscilloscope, and a DAQ module transferring data to a PC. In line with the literature, the circuit was tested using a set of resistive loads. A photograph of the AHCS circuit board is shown in Figure 5b, with its main stages labelled.

## 3. Results and Discussion

### 3.1. Frequency Response

**Figure 6:**
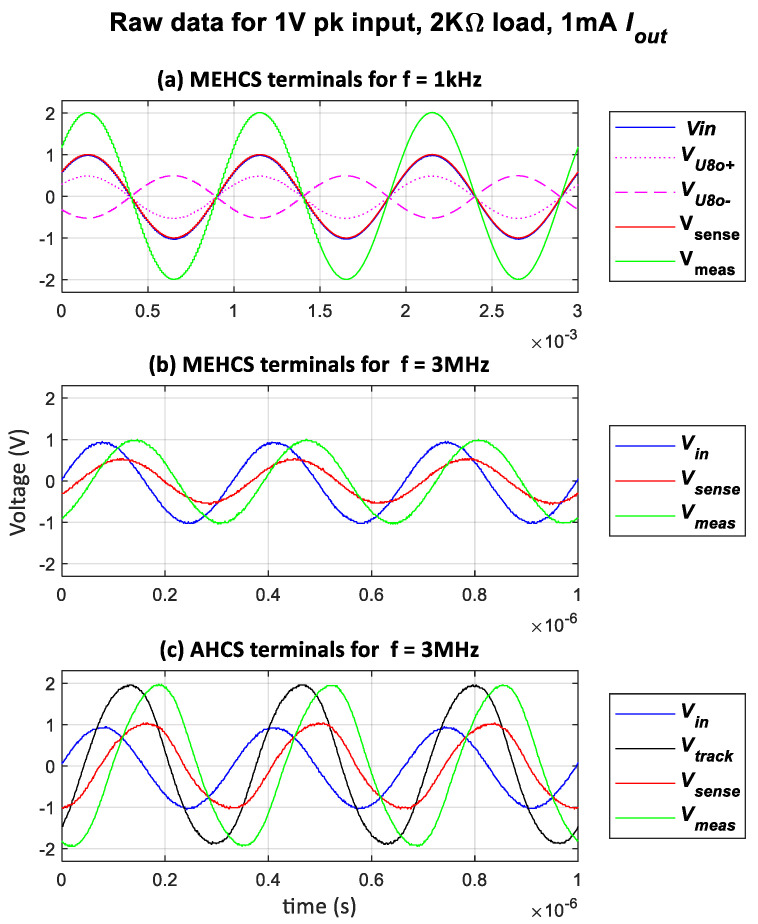
Raw data: input and output voltages of the system in open and closed loop for a 2 kΩ load: (**a**) MEHCS for f = 1 kHz, (**b**) MEHCS for f = 3 MHz, and (**c**) AHCS for f = 3 MHz.

The raw data presented in Figure 6 for the open-loop configuration (MEHCS) at 1 kHz for a load of 2 kΩ load and an output current *I*_out_ of 1 mA indicate that the amplitudes of *V_sense_* and *V_meas_* were respectively 1 V and 2 V, as expected. However, as shown in Table 1, their amplitudes decline at higher frequencies due to the degradation of output impedance, as described in the literature.

This is shown in Figure 6b for 3 MHz, where *V_sense_* ≠ *V_in_* and *V_sense_*, *V_meas_* reached approximately half of their nominal values, indicating a reduction of approximately 50% in the amplitude of the generated current. However, when the AGC was enabled, the multiplier output amplitude *V_track_* increased to compensate for the observed loss in *I_out_*. This compensation continues until *V_sense_* = *V_in_*. At this point, the integrator ceases to charge, and *V_track_* stabilises at its final value. The impact of the Automatic Gain Control (AGC) mechanism of the AHCS is demonstrated in columns 3 and 5 of Table 1. It is also illustrated in Figure 6c, for the same input as in Figure 6b, where both voltages are restored very close to their nominal amplitudes in Figure 6a.

**Figure 7:**
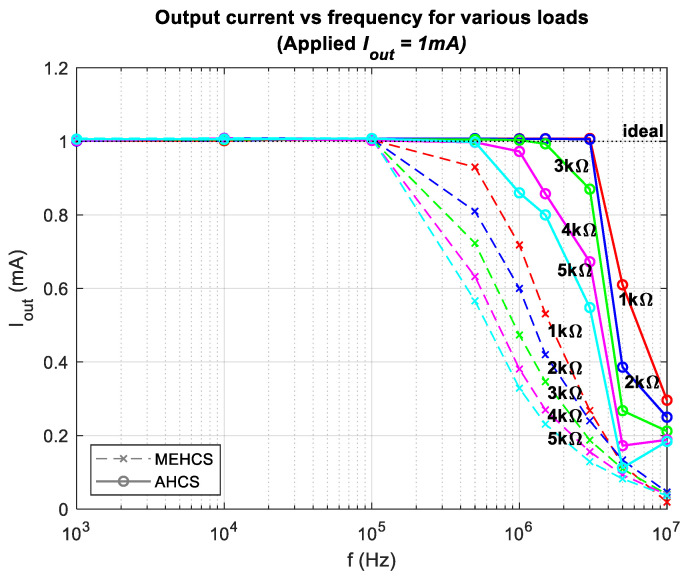
Output current vs. frequency for various loads for MEHCS and AHCS.

Figure 7 shows the output current as a function of frequency derived by dividing the measured *V_sense_* with *A_V_sense_* across loads ranging from 1 kΩ to 5 kΩ. Measurements were taken with an applied voltage of 1 V, with and without the AGC enabled. It is evident that the injected output current of AHCS remained stable at 1 mA over a wider bandwidth than with MEHCS. This was particularly evident with a resistive load of 1 KΩ, where a bandwidth of 3 MHz was attained, in contrast to MEHCS, which achieved acceptable accuracy (≤1% error) only up to 100 KHz for the same load. 

**Figure 8:**
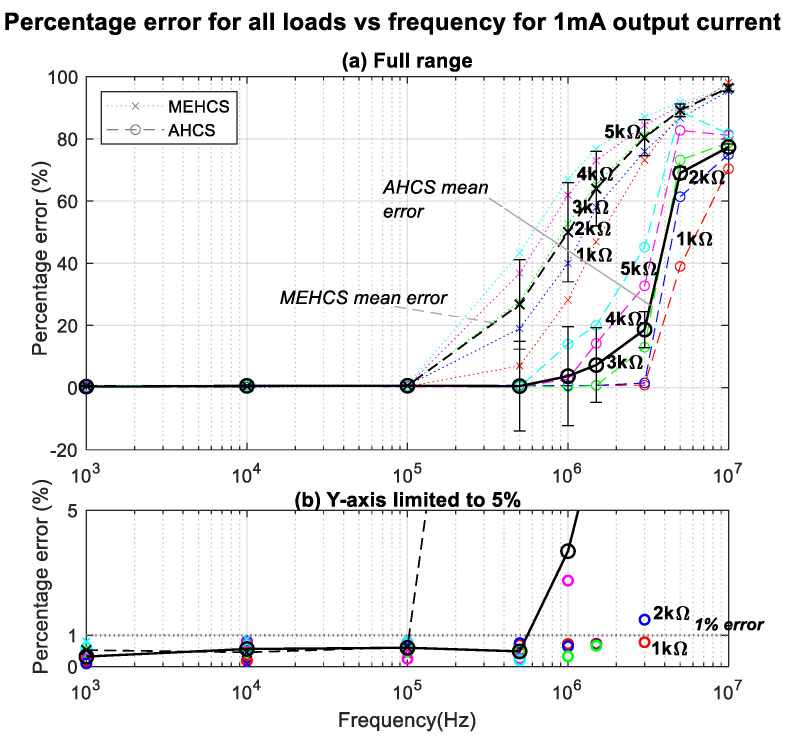
(**a**) Percentage errors of 1 mA output current for MEHCS and AHCS for all loads at all frequencies. (**b**) Same as (**a**) scaled to low error values (up to 5%), highlighting the performance of AHCS for 1 kΩ.

This is better illustrated in Figure 8, where the percentage errors of the trends in Figure 7 are presented. The figure illustrates all output current errors for loads ranging from 1 kΩ to 5 kΩ and the resulting mean error across all loads for all frequencies for the two systems. Significant errors from 100 KHz and above were observed from MEHCS compared to AHCS, for which output current decline (≥1%) occurred only for higher loads above 500 KHz. It could be seen that AHCS demonstrated significantly improved accuracy over a much wider bandwidth compared to the conventional MEHCS, especially for a 1 KΩ load, the load for which the highest reported bandwidth of 1 MHz was shown to be achieved with a 1.5% error in [22]. AHCS exhibited a sub-1% error for up to 3 MHz, i.e., *a significantly lower error for three times the bandwidth, to our knowledge never reported before.* With the same load, MEHCS recorded a 7% error at 500 KHz and 73% error at 3 MHZ. An error just above 1% was achieved for the same bandwidth of 3 MHz for a 2 KΩ load. It can be observed from Figure 7 and Figure 8 that the AHCS performed better and accurately up to 1 MHz for all the loads and up to 3 MHZ for 1 KΩ.

**Figure 9:**
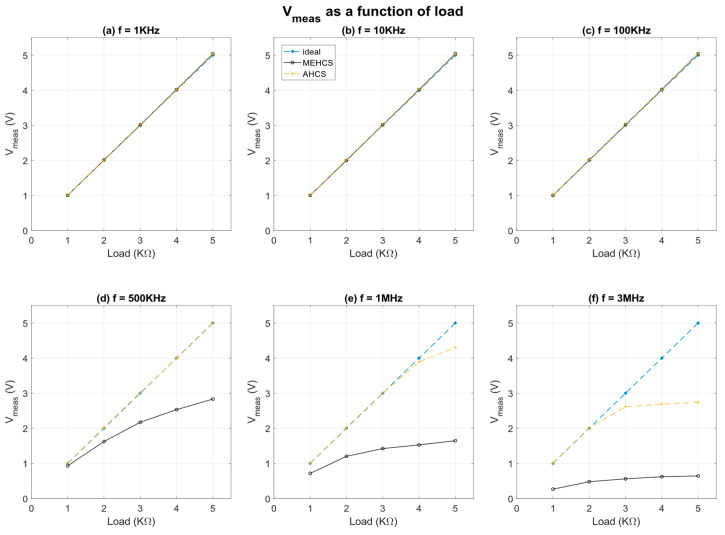
Measured output voltage of MEHCS and AHCS compared to the ideal result as a function of load at frequencies from 1 kHz to 3 MHz with *I_out_* = 1 mA. The legend for all traces is located at the top of subplot (**b**).

Figure 9 illustrates *V*_meas_ for MEHCS and AHCS as a function of load for specific frequencies. The data of Figure 7 indicate that MEHCS exhibits inaccuracy above 100 kHz, and this is clearly illustrated in Figure 9 for all loads 500 kHz and above. This limitation in bandwidth hinders the reliable assessment of bioimpedance tissue properties for a wide range of samples when using MEHCS. In contrast, the implementation of AHCS compensates for these limitations and delivers well-manifested improvement in bandwidth performance. The voltage responses obtained using AHCS remained accurate for all loads up to frequencies of 500 kHz. 

### 3.2. Performance for Smaller Output Current

This expanded bandwidth capability enables precise measurements of impedance across a broader range of frequencies in the β-dispersion, as mentioned previously. In addition to the above, and similarly to the previous figures, Figure 9 also illustrates that AHCS exhibits exceptional accuracy even at higher frequencies, specifically up to 3 MHz, for loads up to 2 kΩ. This performance is noteworthy as it ensures reliable voltage responses and accurate bioimpedance measurements, even when these involve higher load tissues and at high frequencies, well beyond the capabilities of conventional MEHCS.

Designers of bioimpedance instrumentation prioritise output current amplitudes in the mA range, to maximise the signal strength relative to noise and thus improve impedance measurement reliability. Nevertheless, in specific applications, e.g., in cell culture experiments, or for high-contact impedance microelectrodes, sub-mA currents are necessary [31]. However, existing designs like CTVF-MEHCS cannot supply sub-mA currents [22]. 

**Figure 10:**
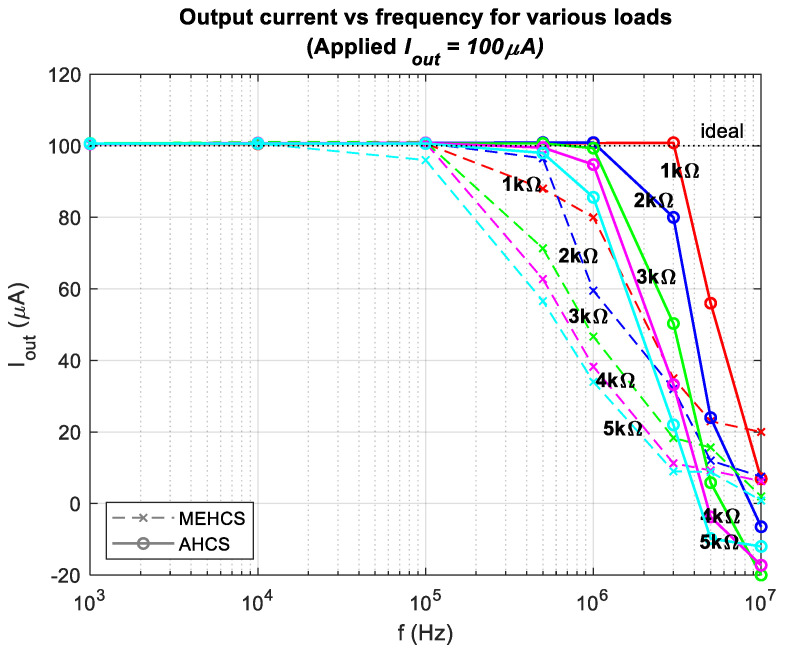
Output current of 100 µA as a function of frequencies for both MEHCS and AHCS.

**Figure 11:**
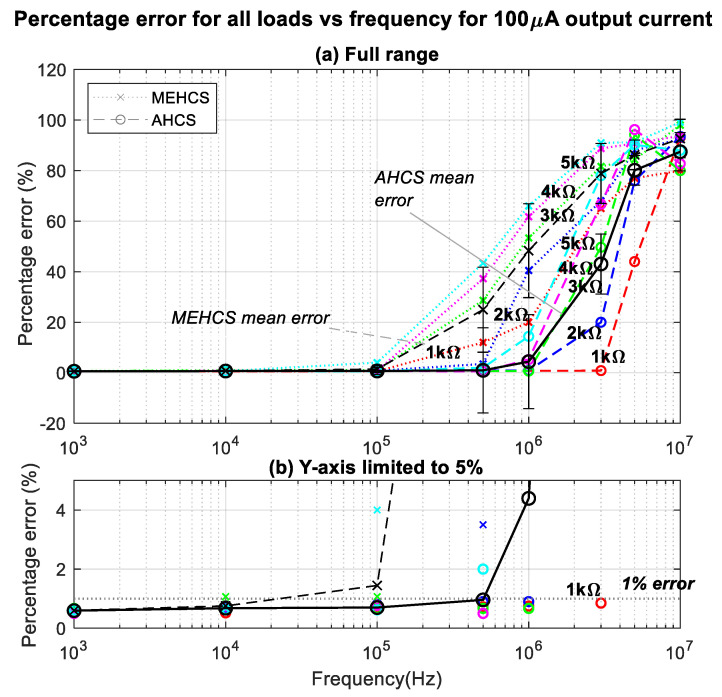
Same as Figure 8 but with sub-mA *I*_out_. (**a**) Percentage errors of 100 µA output current for MEHCS and AHCS for all loads at all frequencies. (**b**) Same as (**a**) scaled to low error values (up to 5%), highlighting the performance of AHCS for 1 kΩ.

Figure 10 was obtained using the same procedure as Figure 7, but with an output *I*_out_ of 100 µA, achieved by setting *V_in_ = 100 mV* for the same transconductance as described in Equation (11). The frequency response in Figure 10 showed a similar trend to that observed in Figure 7 and was further analyzed in Figure 11.

Figure 11 illustrates that AHCS enhances performance even for sub-mA currents. The accuracy and bandwidth results for AHCS are comparable for both 1 mA and 100 µA, as evidenced by the percentage error graphs in Figure 8 and Figure 11, respectively, where the output current error is below 1% for 3 MHz for a load of 1 kΩ. In contrast, while with 1 mA *I*_out_ MEHCS achieved less than 1% percentage error for all loads at 100 kHz (Figure 8), Figure 11 indicates that when sourcing 100 µA at the same frequency, errors up to 4% are observed. 

### 3.3. Output Impedance—Fully Differential Measurement

**Figure 12:**
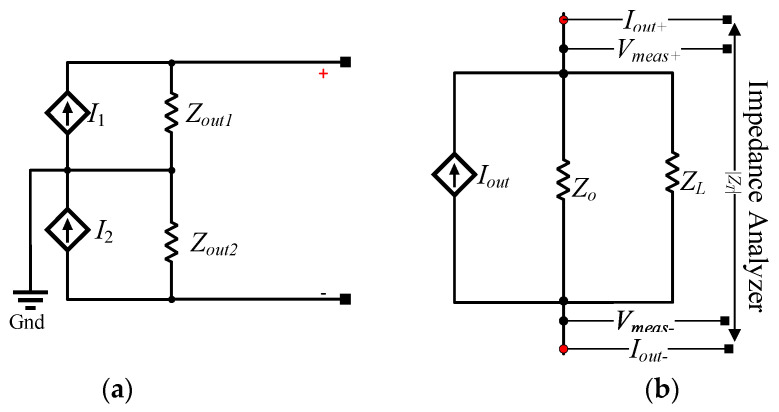
(**a**) Equivalent MEHCS output impedance circuit represented by two single-ended HCS output impedances. (**b**) Measurement configuration used here for determining the output impedance of AHCS.

The performance of the AHCS, demonstrated for a range of frequencies and loads, is based on the AGC essentially adjusting *I_out_* to compensate for output current losses due to output impedance decay with frequency. Still, the measurement of the system’s output impedance over the full bandwidth is of interest for comparison with prior state of the art. Assuming a high op-amp open-loop gain and very low and equal resistor tolerances, the minimum output impedance $Z_{O,min}$ for a single-ended MEHCS can be calculated as in Equation (13) [20,21]:

(13) $$Z_{O,min}(\gamma )=\frac{R*R_{h5}}{2\gamma (R+R_{h5})}$$

assuming that $R_{h1}=R_{h2}=R_{h3}=R_{h4}=R$. The factor *γ* represents resistor tolerances. Measuring the output impedance of MEHCS is a complicated procedure and often inaccurate. This is because the output impedance is conventionally derived through measurements between one output terminal and the ground, in accordance with modelling the circuit as two single-ended HCS current sources in series, as shown in Figure 12a.

As such, the total impedance is then calculated as twice the measured single-ended output impedance [24], represented in Figure 12a by *Z_out_*_1,2_. According to [22], this can lead to errors.

**Figure 13:**
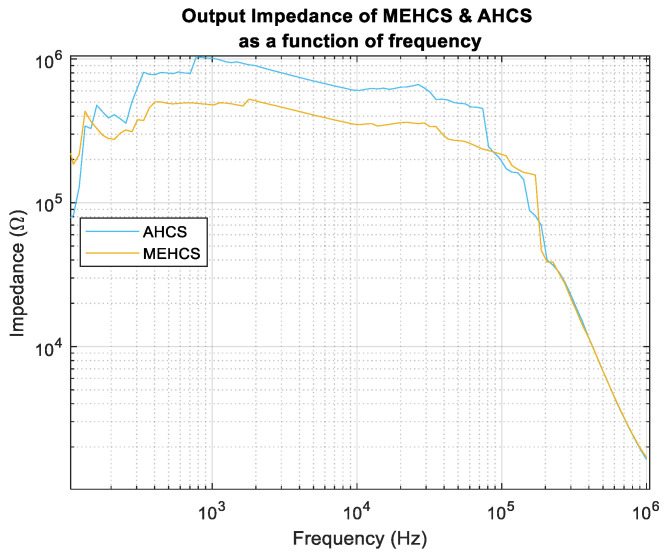
Output impedance as a function of frequency for MEHCS and AHCS.

Here, we initially attempted to measure the full differential *Z_O_* using the E4980A precision LCR meter/impedance analyser, but the measurement was erroneous in low frequencies due to the MEHCS/AHCS output stage essentially connected as an open circuit with the power on. Therefore, a different approach was adopted to achieve a more accurate measurement. A known, well-characterised resistive load, *Z_L_* = 999 Ω, was connected at the output of the MEHCS/AHCS to ensure the output stage operated as a closed-circuit Norton equivalent, and the input to the system was grounded. Using the E4980A, a measurement of $Z_{T}=Z_{o}||Z_{L}$ was carried out from 20 Hz to 1 MHZ, as illustrated in Figure 12b. This was performed with both the AGC disabled (MEHCS) and enabled (AHCS), allowing for the output impedances of both MEHCS and AHCS to be derived from Equation (14) and generating the data for Figure 13.

(14) $$Z_{O}=\frac{Z_{T}Z_{L}}{Z_{L}-Z_{T}}$$

It can be observed from the results in Figure 13 that both MEHCS and AHCS exhibited a similar trend, with AHCS exhibiting higher output impedance at lower frequencies, up to 100 kHz. 

### 3.4. Tracking Speed

**Figure 14:**
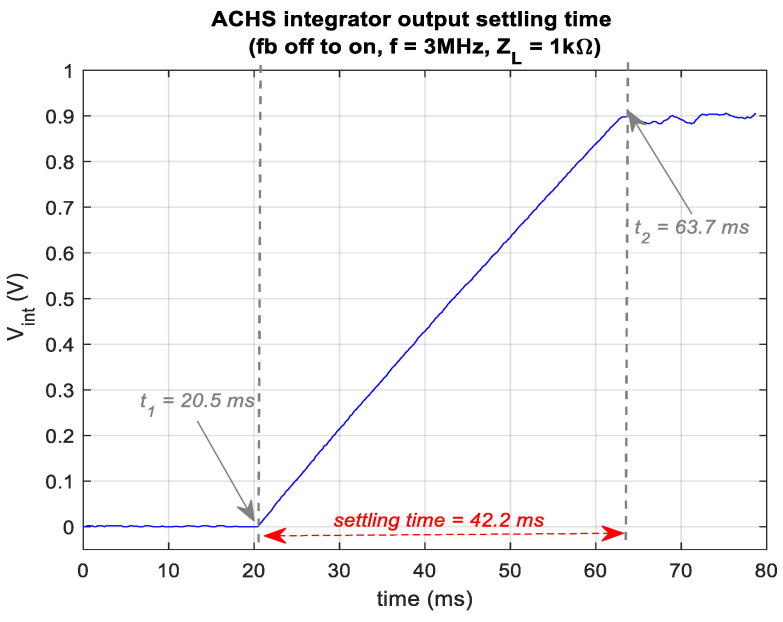
AHCS settling time at its highest operating frequency (3 MHz) with 1 kΩ load when transitioning from fb off (MEHCS) to fb on. The integrator output is baselined to zero.

The response time of HCS-based designs mostly depends on the settling time of the op-amps used (in the absence of feedback capacitors). ACHS exhibits longer response time following abrupt input amplitude changes because of the AGC’s integrator (here chosen to be τ = 12 ms, which is sufficiently lower than our lower injected signal frequency of 1 kHz). In Figure 14, the integrator’s settling time was measured to be 42.2 ms at maximum *V*_in_ bandwidth (3 MHz) with a typical load (1kΩ), with the feedback off and then on. It is noteworthy that this may not be a significant concern in most bioimpedance applications. Exceptions include applications where fast changes need to be monitored (e.g., plethysmography [32,33]) or where specific frame rates across multiple channels are required (e.g., electrical impedance tomography (EIT [34]).

### 3.5. Noise

**Figure 15:**
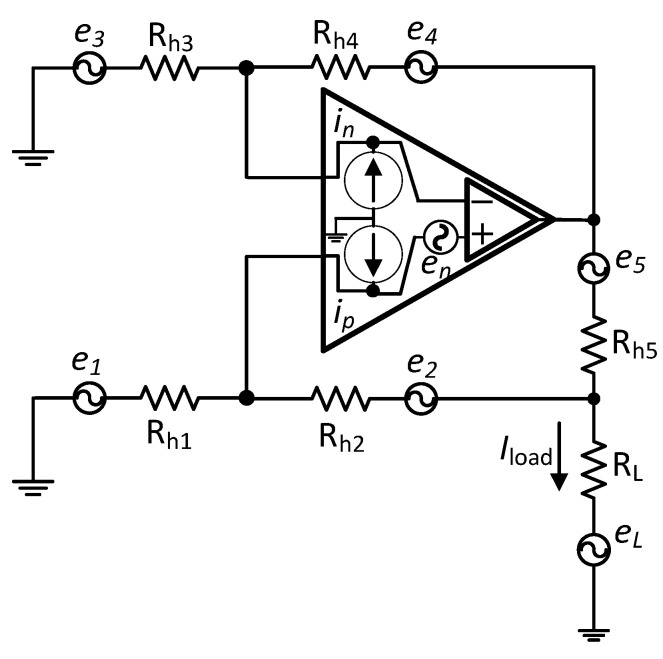
Noise analysis of single-ended Howland current source. $e_{1-5},e_{L}$ are thermal noise spectral densities due to resistors $R_{h1}to\;R_{L}$. $e_{n}$, $i_{n,p}$ are noise voltage and noise current spectral densities of the op-amp.

As mentioned previously, MECHS requires Rh1–4 to be in the order of 0.1–1 MΩ, which results in increased thermal noise. During our performance analysis of AHCS, it became apparent that the AGC improving output current accuracy over a higher bandwidth independently of the system’s output impedance allows for the use of lower value resistors in the MECHS sub-stage. This reduces the overall noise exhibited at the output node. Error sources in MEHCS (Figure 15) can be identified through the analysis of half of the circuit (single-ended HCS with grounded load). The noise sources detailed in the figure translate to output- and input-referred RMS noise voltages, En,out and En,in, respectively.

$E_{n,in}$ has three components (Equation (15)): the internal op-amp voltage noise $E_{nv}\;$(not considered here, as it is not affected by the size of *R_h_*_3_ and *R_h_*_4_); the internal current-introduced noise $E_{ni}$; and the resistor-related noise (thermal) $E_{nr}$ (Equation (17)).

(15) $$E_{n,in}=\sqrt{E_{nv\;}^{2}+E_{ni}^{2}+E_{nr}^{2}}$$

(16) $$E_{ni}=\sqrt{({I_{n}R_{a})}^{2}+{{(I}_{n}R_{b})}^{2}}$$

(17) $$E_{nr}=\sqrt{{\left(E_{nr\_a}\right)}^{2}+{\left(E_{nr\_b}\right)}^{2}}$$

and

(18) $$E_{nr\_a,b}=\sqrt{4kTR_{a,b}{BW}_{n}}$$

where BW_n_ = noise bandwidth of the op-amp, k = 1.38 × 10^−23^ J/K (Boltzmann’s const.); *I*_n_ = RMS noise current, and T = temperature (K). $R_{a}$ and $R_{b}$ correspond to the two feedback branches, as follows:

(19) $$R_{a}=R_{h3}∥R_{h4}=\frac{R_{h3}R_{h4}}{R_{h3}+R_{h4}}$$

(20) $$R_{b}={((R}_{h5}∥R_{L})+R_{h2})∥R_{h1}$$

Consequently, the total output-referred RMS noise voltage is

(21) $$E_{n,out}=A_{v\_CL}E_{ni}$$

The contribution of the noise sources to the output current is

(22) $$\;I_{load,TN}=\sqrt{\left(\frac{{E^{2}}_{nv}+{E^{2}}_{ni}+{E^{2}}_{nr}}{{R^{2}}_{h5}}\right)}$$

**Figure 16:**
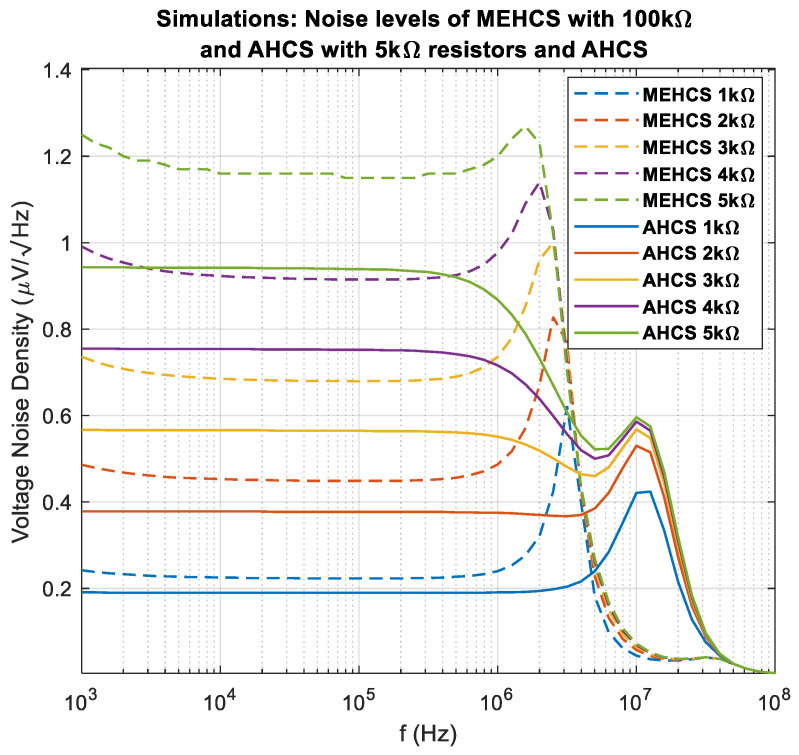
Simulation: voltage noise across different loads for single-ended MEHCS with 100 kΩ *R_h_*_1_, *R_h_*_3_, *R_h_*_4_, and (*R_h_*_3_ + *R_h_*_5_) and AHCS when using 5 kΩ instead. MEHCS does not work with resistor values lower than 100 kΩ, while AHCS does.

**Figure 17:**
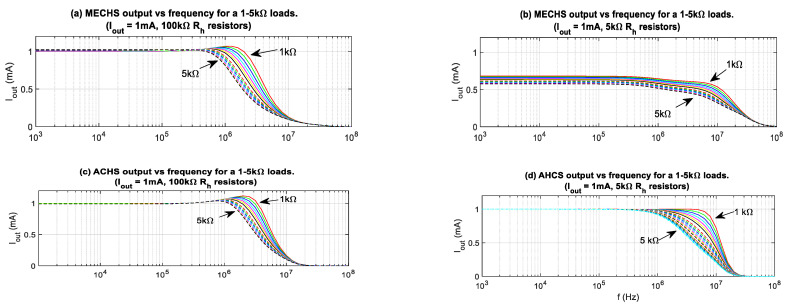
Simulation: the effects on *I*_out_ of lowering *R_h_*_1*–*4_ from (**a**) 100 kΩ to (**b**) 5 kΩ in single-ended MEHCS. (**c**,**d**) Using 100 kΩ and 5 kΩ *R_h_*_1–4_, respectively, for a single-ended AHCS.

Figure 16 demonstrates the noise spectrum for MEHS and AHCS for different loads. MEHCS is demonstrated with 100 kΩ *R_h_* values, as it is not operational for lower values, while lowering *R_h_* values to 5 kΩ for the AHCS (still operational in Figure 17) allows for improved noise performance, with AHCS achieving 17% lower noise for a 1 kΩ load and 19% lower noise for a 5 kΩ load within the bandwidth of interest.

Equations (16), (17), and (22) further indicates that selecting large values for *R_h_*_3_ and *R_h_*_4_ increases the noise levels in the MEHCS design. Attempting to lower the value of the *R_h_* resistors from 100 kΩ (as used in the experiments) to 5 kΩ results in *I*_out_ degradation for the MEHCS. This is shown in the respective simulated *I*_out_ frequency responses in Figure 17a, where MEHCS delivers the nominal current of 1 mA, and Figure 17b, where the current delivered is lower than the nominal and changes for different loads. Figure 17c indicates that the same reduction in *R_h_* values does not affect the *I*_out_ frequency response of AHCS, which is 1 mA independent of load. Simulations were carried out in single-ended half-circuits with grounded loads.

### 3.6. Component Tolerance

The precision foil resistors used for R_h1_–R_h5_ and R_sense_ have a TCR (temperature coefficient of resistors) of ±2.0 ppm/°C and a tolerance of ±0.01%. Nominal and calculated values for a 1 kΩ resistance of the same type as R_sense_ are presented in Table 2 for temperatures −20 °C, 0 °C, and 40 °C.

The impact of temperature on ACHS components and particularly the current sensing resistor R_sense_ was further examined through LTSpice simulations, spanning temperatures from −20 °C to 40 °C, with a temperature coefficient set to the specified 2.0 ppm/°C as per the precision foil resistor datasheet. The reference temperature was set at 27 °C.

(23) $$R_{a}=R_{ref}(1+TCR(T_{a}-T_{ref}))$$

**Figure 18:**
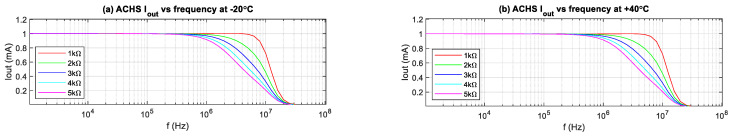
Percentage errors of ACHS at extreme temperature variations. (**a**) Simulated at −20 degrees centigrade and (**b**) Simulated at 40 degrees centigrade.

Replacing the R_h_ resistors with R_α_, calculated using Equation (23), AHCS was once more assessed by generating its frequency responses for a range of loads (Figure 18). The graphs demonstrate that extreme temperature variations do not have a significant effect on the system’s performance.

## 4. Performance Overview

**Figure 19:**
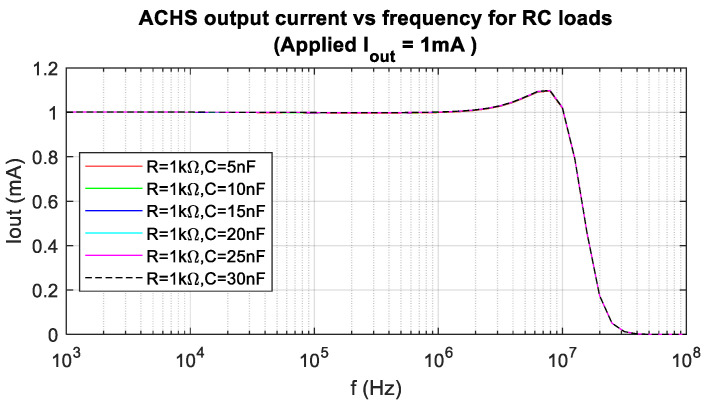
Simulation: AHCS output current vs. frequency for RC loads (applied *I*_out_ = 1 mA).

The overall performance of the AHCS system is summarised in Table 3, in comparison with six other architectures of AC current sources, including an ASIC approach [18] featuring an OTA-based design. The AHCS architecture achieves a higher bandwidth than the ASIC for a less than 1% output current amplitude error. It also achieves outputs as low as 100 µA, lower than the 500 µA reported in [16]. The total harmonic distortion (THD) was measured to be less than 0.2% for 2 mA p-p output current. The system was also simulated with RC loads (Figure 19) comprising a 1 kΩ resistor in parallel with capacitors ranging from 5 nF to 30 nF. The bandwidth exhibited was stable for all loads.

## 5. Conclusions

Conventional fixed-gain AC current source designs, commonly used in bioimpedance applications, exhibit output current amplitude decline at higher frequencies, limiting their bandwidth to around 100 kHz. Modified Enhanced Howland Current Source (MEHCS) design variants have shown promise with 1 MHz bandwidth and low output current errors of 1.5%. However, there remains a need for achieving less than 1% current errors, higher bandwidths, and operation with sub-mA currents in various bioimpedance applications, a feat not yet attained by existing optimised designs.

In this paper, we introduced the Adaptive Howland Current Source (AHCS), which surpasses current systems by achieving sub-1% output current error over a bandwidth three times greater than the highest reported, including performance at smaller (100 µA) currents. We presented a discrete-component realisation in line with most of the relevant literature, and its performance surpassed even ASIC realisations. AHCS incorporates an Automatic Gain Control (AGC) to adapt input voltage amplitude, autonomously compensating for output current degradation. In contrast to conventional optimisation approaches, AHCS enhances accuracy across a broader bandwidth by dynamically adjusting the current driver reference voltage based on actual injected current output, offering flexibility for swept frequency applications and high performance for low amplitude currents, as needed in cell culture experiments.

AHCS’s strength lies in maintaining constant amplitude AC current outputs with great accuracy over a significantly wider bandwidth, albeit with trade-offs such as increased complexity, power consumption, system size, and settling time. Despite these considerations, AHCS’s wide bandwidth operation offers substantial performance improvements, making it conducive for highly accurate bioimpedance measurements at high frequencies. The envisioned applications encompass extending bioimpedance monitoring methods beyond research settings into mainstream clinical practice, assessing conditions like skin and oral cancer.

## Figures and Tables

**Table 1:** MEHCS and AHCS measured peak outputs.

*f* (Hz)	*V_sense_* (V)		*V_meas_* (V)	
	MEHCS	AHCS	MEHCS	AHCS
1k	1.018	1.021	2.011	2.002
10k	1.020	1.011	2.002	2.016
100k	1.032	1.012	2.014	2.015
500k	0.861	1.021	1.620	2.015
1M	0.600	1.016	1.201	2.013
3M	0.211	1.001	0.480	2.011

*Load = 2 KΩ. R_sense_ = 100 Ω. IA gains: A_V_sense_ = 10, A_V_meas_ =1. Nominal I_out_ = 1 mA. V_in_ = 1 V*.

**Table 2:** Calculated resistance of the sense resistor (R_sense_) at −20° and 40° extreme temperature variations.

R_ref_ Nominal (Ω)	TCR (ppm/°C)	T_a_ (°C)	R_a_ Measured (Ω)
1000	2	−20	999.906
1000	2	0	999.946
1000	2	40	1000.026

**Table 3:** Main features of AHCS and comparison with other designs.

Architecture	CTVF-MEHCS [22]	EHCS [35]	Differential Sinusoidal Current Gen [36]	OTA—Based ASIC [18]	Differential Difference Amplifier (DDA) [37]	OTA and DDA (ASIC) [38]	ACHS (This Work)
Bandwidth < 1% I_out_ err.	≤10 kHz	<100 Hz	90 kHz	≤1 MHz	≤1 MHz	≤200 kHz	≤3 Mhz
THD	--	--	0.81% @ 250 µA_p-p_	0.69% @ 5 mA_p-p_ 0.53% @ 2 mA_p-p_	--	--	<0.2% @ 2 mA_p-p_
Size and complexity	--	--	5 mm × 5 mm	6.18 mm^2^	--	0.21 mm^2^	15.5 cm × 8.5 cm
Max Output Current	2 mA_p-p_	<1.4 A_pk_	350 µA_p-p_	5 mA_p-p_	20 mA	0.7 mA_p-p_	2 mA_p-p_
Output Impedance	3.16 MΩ @ 10 kHz. 1.99 MΩ @ 100 kHz <32 KΩ @1 MHz	--	>100 KΩ @ 90 kHz	665 KΩ @ 100 kHz 372 kΩ @ 500 kHz	<100 K @ 1 MHz	40 @ 30 kHz	1 MΩ @ 1 kHz 200 KΩ @ 100 kHz. <1 kΩ @1 MHZ
Output current accuracy	<1%eror @ 10 kHz <2% @ 1 MHz 68.5% @10 MHz	<0.5% @ <100 Hz	≤1% @ 90 kHz	<1%eror @ 1 MHz	<1%eror @ 1 MHz	--	<1% @ 1 mA @ 3 MHz
Supply Voltage	18 V	±25 V	1.25 V	18 V	---	±0.8	±15 V
Technology	Discrete	Discrete	0.18 µm CMOS	0.6 µm CMOS HV	Discrete	0.65 nm CMOS	Discrete

## Data Availability

The original contributions presented in the study are included in the article, further inquiries can be directed to the corresponding author.
